# Knowledge and practice regarding the behavioural risks of cancer among college students in Ethiopia

**DOI:** 10.3332/ecancer.2022.1343

**Published:** 2022-01-13

**Authors:** Foziya Mohammed Hussien, Anissa Mohammed Hassen, Zinet Abegaz Asfaw, Aragaw Yimer Ahmed, Yeshimebet Ali Dawed, Ahmed Hussien Asfaw, Erkihun Tadesse Amsalu, Hamid Yimam Hassen

**Affiliations:** 1 Department of Public Health, College of Medicine and Health Science, Wollo University, Dessie 1145, Ethiopia; 2 Department of Internal Medicine, School of Medicine, College of Medicine and Health Science, Wollo University, Dessie 1145, Ethiopia; 3 Department of Public Health, College of Medicine and Health Science, Mizan-Tepi University, Mizan Teferi 260, Ethiopia

**Keywords:** knowledge, behavioural risk factor, cancer, student, Ethiopia

## Abstract

In recent years, morbidity and mortality due to cancer has been increasing in Ethiopia, putting it among the most important public health issues. Cancer and associated complications can be prevented provided that effective interventions are put in place to control risk factors. Therefore, this study aimed to assess the level of knowledge and practice of preventable behavioural risk factors of cancer. We conducted a cross-sectional study among 200 college students in Northeast Ethiopia utilising quantitative methods of data collection. Data on socioeconomic characteristics, health belief variables, knowledge and behavioural risk factors (alcohol consumption, tobacco smoking, physical activity and dietary practice) were collected. The level of knowledge and practice was summarised using descriptive statistics. To investigate the variation in knowledge and practice across sociodemographic characteristics, we performed Pearson Chi-square test or Fisher’s exact test. The majority (81.0%) of participants was male and 82.0% were in the age group of 18–24 years. More than half (61.0%) of them had poor knowledge about the behavioural risk factors of cancer. Nearly one-third (30.5%) consume alcohol, whereas 16.0%, 18.0% and 20.0%, respectively, smoke tobacco, consume street food and packed-fried snacks daily. Alcohol consumption (*p* = 0.02) and level of vigorous physical activity (*p* = 0.001) were significantly higher among males than females. Alcohol consumption, tobacco smoking and unhealthy dietary practice were remarkably high, whereas knowledge towards the behavioural risk factors was low. Therefore, health education and collaborative action between different sectors are needed to counter the emerging problem.

The trial is registered in ClinicalTrials.gov (https://register.clinicaltrials.gov) NCT04269018.

## Introduction

Improvement in global health has improved life expectancy, enhanced maternal and child health and reduced the spread and consequence of infectious diseases [1]. However, major challenges to human health and development persist, with chronic diseases including cancer still the major cause of death and disability [2, 3]. By the year 2040, the global cancer burden is expected to exceed 27 million new cancer cases per year [4]. Globally, with an estimated 19.3 million cancer incident cases and 10 million deaths in 2020, it is the second leading cause of death next to cardiovascular diseases [5, 6]. Previously, Non-Communicable Diseases (NCDs) including cancer were considered a disease of high-income countries, but now they are also an important public health issue in low- and middle-income countries (LMICs) [6, 7]. Lifestyle changes, rapid urbanisation, cultural transition and an increase in life expectancy could contribute to a rise in incidence [6, 8]. This imposes an enormous burden on the already overwhelmed healthcare system of LMICs since cancer treatment centres are not sufficient to cover the increasing needs of the affected population [9, 10].

Despite a steady increase in cancer morbidity and mortality, it has been largely neglected by the governments of LMICs, international organisations and civil societies due to the existing infectious diseases. As a result, we should not forget the terrible effect on human lives and economic cost which is imposed by cancer [9]. Additionally, p*oor surveillance, research and unresponsive health policies remain a challenge to the prevention of cancer in LMICs* [11]*.*

In Ethiopia, the numbers of cancer cases were estimated to be 77,352 and were responsible for 51,865 deaths nationally in 2020 [12]. Cancers of the breast (20.9%), cervix (9.6%), colorectal (7.8%), leukaemia (5.6%) and Non-Hodgkin lymphoma (4.9%) are the most commonly occurring cancers in the country [12]. In Ethiopia, public health policy gives little attention to cancer prevention and control. Recently, the Federal Ministry of Health launched the National Cancer Control Programme focusing on the multi-sectoral integration of cancer prevention and control [13]. However, the rise in cancer incidence has emphasised that the control practices are still suboptimal.

Risky behaviours including alcohol consumption and cigarette smoking are higher among college students than the general population [14–18]. School-based intervention programmes and policy setting on substance use and other risky behaviours are important to reduce the modifiable risk factors of cancer [17]. Therefore, studies at various levels and contexts are important to insight policy makers on the potential area of interventions.

In Ethiopia, cancer screening, diagnosis and management are sub-optimal and population-based data are limited to few cities [20]. Few studies in the country showed poor knowledge towards risk factors and screening, which were identified as important factors for poor utilisation of cancer screening and other prevention services [21–23]. Thus, investigating the knowledge, perception and practice of the modifiable risk factors of cancers is crucial to develop and implement targeted preventive interventions to halt the growing burden of cancer.

## Materials and methods

### Study setting, design and sampling

A cross-sectional study was conducted to assess knowledge, perception and practice towards the behavioural risk factors of cancer among college students in Dessie, Northeast Ethiopia in July 2021. Dessie town is 401 kms far from Addis Ababa, the capital city of Ethiopia. The commonly cultivated staple foods in the study area are barley, oats, bean and *teff*. There are three public colleges in the city, which provide several academic and training services. All public colleges in the city were included in the study, namely Woizero Siheen Polytechnic College, Dessie College of Teachers Education and Dessie Health Science College. Woizero Siheen Polytechnic College was established in 1930 and currently provides technical and vocational education and training in five different campuses: Main Campus (Siheen Campus), Merho Campus, Hotie Campus, Dawdo Campus and Menen (Vocational Campus). The College has 5,975 trainees in regular and night-shift programmes. The college is also providing short-term, non-formal and in-company training. Dessie College of Teachers Education was established in 1980 and has 1,426 students.

This study is a baseline assessment of a randomised control trial aimed to evaluate the *effectiveness of mobile text messages on knowledge and perception towards cancer and behavioural risks among college students in Northeast Ethiopia.* The trial was registered at ClinicalTrials.gov (https://register.clinicaltrials.gov) NCT04269018 and the protocol was published in PLOS ONE [24]. The sample size was determined using superiority trial design and then a total of 200 participants were enrolled in the study. The sample size was allocated to Woizero Siheen Polytechnic College and Dessie College of Teachers Education based on proportional to the number of students. Students’ identification (ID) was obtained from the registrar’s office of each college and used as a sampling frame. Then, a simple random sampling method was used to select eligible study subjects. Students in the selected public colleges aged 18–35 years with no prior diagnosis of any type of cancer were included, whereas students of Health Science College were excluded as they are expected to know the risks of cancer and preventive mechanisms.

### Data collection and quality control

We captured data using self-administered questionnaires on sociodemographic, knowledge, perception and practice of the behavioural risks of cancer. Knowledge was assessed with 19 questions regarding high-risk groups, risk factors, complications and preventive mechanisms of cancer with ‘yes’, ‘no’ and ‘I do not know’ options. The correct responses were assigned a score of 1, whereas ‘No’ or ‘I do not know’ responses were assigned 0 [25]. The cumulative mean score of knowledge of students was estimated. As a result, those who had scored less than the mean were considered to have poor knowledge and those who had scored greater than or equal to the mean value were considered as having good knowledge [26].

To assess the risk perception of cancer, we obtained data on the health belief model components: Perceived susceptibility towards cancer (e.g. You perceived that you will get risk of developing cancer at any time of your life), Perceived severity of cancer (e.g. ‘Cancer is fatal, and its complication are dangerous/severe in life’), Perceived benefits of adopting behaviours (e.g. ‘Having a healthy nutrition would decrease the probability of any type of cancers’), Perceived barriers to adopting behaviours (e.g. ‘My monthly income is insufficient to take the recommended diet for the prevention of cancer’), Perceived self-efficacy for adopting dietary behaviours (e.g. ‘I am confident that I can prevent cancer through healthy lifestyle’), Internal cues for adopting dietary behaviours (e.g. ‘when knowing the death of any by cancer, it flips me to perform the preventive behaviors’), the items of this subscale were measured on a Likert scale ranging from 1 = ‘Strongly disagree’ to 5 = ‘Strongly agree’ [27].

Physical activity level was measured using the International Physical Activity Questionnaires short form. This contains the frequency and time participants spent doing vigorous physical activity, moderate physical activity, walking and sitting in the last 7 days. Alcohol intake was assessed using the Alcohol Use Disorders Identification Test (AUDIT), a tool adapted from the World Health Organization (WHO) which assessed participant’s consumption in the past 1 month [28]. The frequency and quantity of alcohol consumption were classified into light (1 and 2 drinking occasions per week), moderate (3 and 4 drinking per week) and heavy (5 and more drinking per week) [29]. Moreover, tobacco use was measured using a series of questions adapted from the WHO, which assessed the duration, quantity and frequency of smoking. Current smokers were defined as those who smoked 100 cigarettes in their lifetime and currently smoke cigarettes, whereas ever-smokers represent those who smoked at least 100 cigarettes in their lifetime but quit smoking during the survey [30]. Smokers were classified as light (smoking 1 to 10 cigarettes sticks per day), medium (smoking 11 to 20 cigarettes per day), heavy (smoking 20–40 cigarettes per day) and very heavy smokers (smoking on average more than 40 cigarettes per day) [31]. Dietary habit was measured using a food frequency questionnaire adapted from the Dietary Assessment Primer which was developed by the USA National Cancer Institute and then modified based on the study objectives and local staple foods [32]. The type and frequency of food items scaled with Never Consumed (NC) = 1, Once Per Month (OPM) = 2, Two to Three per Month (TTM) = 3, Once per Week (OW) = 4, Two times per Week (TW) = 5, Three to Four times per Week (TFW) = 6, Five to Six times per Week(FSW) = 7, every day (ED) = 8 were assessed. One month food consumption pattern with foods consumed either inside or outside the home and greater than one tablespoon were measured. We used principal component analysis (PCA) to generate diet scores and classify food consumption patterns. As a result, study participants were categorised into three groups such as poor healthy eating habit, medium healthy eating habit and good healthy eating habit.

The data were collected by newly graduated BSc nurses. After we received an official letter from Wollo University, each college’s administrator and the registrar were contacted to confirm their willingness and to obtain a student list with ID number. We approached 200 students and fortunately, all of them gave their consent to participate in this study.

The validity of the items was checked by an expert panel of specialists in health education, oncology and nutrition. They provided intellectual judgment on the necessity and relevance of the scaling items. The questionnaire was first prepared in English language and translated to Amharic and again re-translated into English by another person to check for consistency. About 10% of the questionnaire was pretested then internal consistency was checked (Cronbach’s alpha = 0.80). To control the data quality, all data collectors were trained for 2 days on the aim of the study, methods and how to take informed consent and administer the questionnaire. Moreover, supervisors closely monitored the data collection process.

### Data analysis

Upon checking for completeness and consistency, data were coded and entered using EpiData version 3.1 (https://epidata-entry.software.informer.com/3.1/). Then, the clean data were exported to a free statistical software R version 4.0.3 for further processing and analysis. Descriptive statistics including absolute and relative frequencies, and cross-tabulations were used to summarise categorical variables. For continuous variables, the normality of the data was checked and then summarised using either mean with standard deviation (SD) or median with interquartile range (IQR). PCA was computed to generate diet score. To investigate the variation in cancer risk knowledge and behaviour across sex, we used Pearson Chi-square statistic or Fisher’s exact test.

## Results

### Sociodemographic characteristics

The mean age of participants was 21.6 years (SD: 3.3), and 82.0% were within the age group 18–24 years. Majorities (81.0%) were male, 87.5% were single, 54.5% were Muslims and 89.4% were from the Amhara region. The median family size was 5.5 (IQR: 4–7) persons per household. The median household monthly income was 7,000 Ethiopian birr (1 ETB ~ 0.025 USD) (IQR: 4,875–10,000) (Table 1).

### Knowledge and perception towards the behavioural risk factors of cancer

Out of a maximum of 19, the mean knowledge score was 6.6 (SD = 3.8) and 61.0% of students had poor knowledge on the behavioural risk factors of cancer. The average score of perceived susceptibility and severity was 11.0 (SD = 3.5) and 21.2 (SD = 5.7), respectively. The mean score of perceived benefit was 24.4 (SD = 6.3), whereas the score for the perceived barrier was 23.36 (SD = 6.32). Similarly, the mean score of cues to action and self-efficacy was 17.9 (SD = 5.2) and 28.7 (SD = 7.6), respectively.

### Behavioural risk factors of cancer

Thirty-two (16.0%) students reported as current smokers, of which 93.7% smoke daily. Twenty-two (68.8%) are light smokers and 37.5% started smoking within the age of 16–20 years. Fourteen (7.0%) have smoked cigarettes in the past daily, but stop smoking currently. Nineteen (9.5%) students used smokeless tobacco or someone smoked within their presence. Nearly one-third (30.5%) of students consumed alcohol in their lifetime, of which 90.2% drank 1 month preceding the survey. Nearly one-fourth (23.6%) were light alcohol drinkers, while 20% usually used alcohol with meals (Table 2).

One month preceding the survey, 86 students were engaged in vigorous physical activity, of which 37.2% practised frequently (5–7 days per week) and 8.1% stayed for a long period (5–7 hours per day) in vigorous activity. Above two-fifths (40.5%) of them were involved in moderate physical activity, of which 45.7% performed 3–4 days per week and 85.2% stayed 1–3 hours per day. Half (50.5%) of them walked or used bicycles for a minimum of 10 minutes, of which 44.6% walked frequently (5–7 days per week) and 82.2% stayed for 1–3 hours per day. Majorities (67.0%) stay 1–3 hours per day sitting, whereas 64.8% sleep 7–10 hours per day (Table 3).

The mean diet score was 63.6 (SD = 10.7) with a minimum value of 46.0 and the maximum value of 102. One-third (34.0%) of study subjects had poor healthy eating habits and 32.5% had good healthy eating habits 1 month preceding the survey. Majorities (82.5%) consumed a cereal-based diet daily within a month prior to the survey. Nearly half (47.2%) consumed legumes and 22.5% consumed nuts and vegetable oils daily. Twenty-one (10.5%) and 6.5%, respectively, consumed fruits and vegetables daily. Nineteen (9.5%) consumed flesh or organ three to four times per week, while none of the participants consumed fish and seafood twice per week within a month prior to the study. Only 2.5% consumed eggs and 5.5% used dairy products daily. Thirty-six (18.0%) students consumed street foods and 4.5% used sugars and sweets more than one tablespoon daily. Nearly half (48.5%) and 20.0%, respectively, consumed salt and fried snacks daily. About 7.0% of study participants used fat and palm oil, and 16.5% used coffee and tea daily within a month preceding the survey (Table 4).

### Sex distribution with knowledge and behavioural risk factors of cancer

Table 5 summarises the disparities in knowledge, perception and behaviour across sex. About 100 (61.7%) of males and 57.9% of females had poor knowledge of the behavioural risks of cancer, but the difference was not statistically significant (*p* = 0.66). Above half (51.2%) of males and 55.3% of females had poor dietary practice a month prior to the survey with no significant sub-group difference (*p* = 0.65). Twenty-nine (17.9%) males and 7.9% females were smokers with no sub-group difference (*p*-value = 0.14). The prevalence of alcohol drinking was higher among males (30.9%) than females (13.2%) and the difference was statistically significant (*p* = 0.02). More males (48.8%) were engaged in vigorous physical activity than females (18.4%) and the difference was statistically significant (*p* < 0.001). Sixty-nine (42.6%) males and 31.6% females were engaged in moderate physical activity. The majority (71.2%) of males and 52.6% females spent 1–3 hours sitting, whereas 68.4% males and 50.0% females sleep 7–10 hours per day (Table 5).

## Discussion

Cancer prevention is key to tackling the incidence, decreasing mortality and reducing the social and economic burden that is imposed by cancer. Over half of all cancers could be prevented if appropriate measures are taken on the behavioural risk factors of cancer [19]. Hence, behaviour change is possible, powerful and cost-effective in the cancer prevention continuum [33]. Therefore, this study summarises the knowledge level and prevalence of behavioural risk factors among adults which help to ease the focus of interventions.

The majority of study participants had poor knowledge on the behavioural risk factors of cancer. This finding is consistent with several studies in different parts of the world [34–36]. Awareness of the modifiable risk factors is important and could be the primary strategy to improve cancer prevention and control [38]. Moreover, this might help people understand the potential health consequences of their actions and encourage them to make behaviour changes [37]. Therefore, it is important to improve public health messages about how lifestyle risk factors impact the chances of developing cancer.

In the National Cancer Control Programme of Ethiopia, the Ministry of Education was assigned to incorporate healthy lifestyles in the curriculum of college students [13]. However, healthy lifestyles and other cancer prevention programmes have yet to be incorporated in non-healthy students. Therefore, all stakeholders should be actively involved, committed and timely coordinate activities to successfully implement the programme and reduce the national burden of cancer.

In the present study, fruit and vegetable consumption is low, which increases the risk of various diseases including cancer [39–42]. It is supported by other studies done in Ethiopia [43, 44]. This is due to the imbalance between the price of fruits and vegetables and affordability by the community [43]. Therefore, identifying this individual could help them find ways to include more fruit and vegetables in their diets [40]. Additionally, increasing the production and access of fruits and vegetables will help to subside the price and stabilise the market.

The majority consumed street foods and packed fried snacks daily, hence unhealthy foods persuasively increased the risk of cancer [41]. This finding is supported by a study done in Addis Ababa in which adolescents preferred packed and street snacks than home-prepared food [45].

Food preference, poor nutritional knowledge and food safety were the possible explanations for choosing packed fried and street foods [45]. Moreover, the price of fruits, vegetables and animal sources of foods was higher than unhealthy street foods, which are easily and widely available at a cheap cost [46]. Therefore, keeping the safety of foods all over the value chain could increase the access and year-round availability of healthy foods. Additionally, taxing unhealthy foods, subsidising the cost of healthy foods and providing fixed costs to staple fruits could help to stabilise the market. Awareness creation on the risk of unhealthy foods and restricting from college compounds could help to reduce the consumption of unhealthy foods and give value to healthy foods. The food services in colleges of Ethiopia particularly Dessie city are predominantly fried or canned snacks and sugary drinks. Thus, the college administration should consider establishing rules for college catering services to serve healthier food.

We found that males used more alcohol as compared to females which is convincingly linked with a large increase in cancer [33, 41, 42, 47]. It is consistent with previous studies done in Ethiopia reportedly, male sex was found to be the independent predictor of alcohol consumption [48, 49]. In Ethiopia, the beverage industries have increased leading to a significant increase in alcoholic beverage production and mass media advertisements coupled with many promotional awards exposing males to consume much alcohol. In the Ethiopian context males are more active on social media and spend more time in the outdoors than females. Therefore, awareness creation activities for college students are important to minimise the risk of cancer.

The physical activity level in the present study was low, which increases the risk of cancer [41, 42]. Similarly, male sex was significantly associated with vigorous physical activity in line with a study done in Debre Birhan, Ethiopia [50].

This might be due to a lack of female-friendly facilities and gender role variation, hence females engaged more in routine homework activity and child caring practice which makes them less active than males [50]. However, males are more likely to be involved in work related activities such as farming, biking and digging than females. In general, low availability of physical exercise facilities and playgrounds and high subscription fee are the main barriers to physical activity in Ethiopia [50, 51]. Therefore, the sport and youth office should work in collaboration with the health and women affairs office administrators for fulfilling and making facilities (gymnasium and playground) accessible to the public. Moreover, they have to make all the facilities girls friendly and empower women to be physically active.

The present study should be interpreted considering the following limitations. Firstly, we rely only on subjective assessment of most of the outcome variables including physical activity level, alcohol consumption and smoking, which might lead to underestimation of such behaviours. Nevertheless, the use of self-administered questionnaires could reduce the social desirability bias. Secondly, we didn’t collect a few important variables such as nutritional status, which take a part in the behavioural risk factors of cancer. Thirdly, to assess the dietary habit, we relied on food groups instead of food items, in which specific food items could vary in nutritional value and the actual intake of nutrients was not estimated and weighted. Lastly, this study focused on college students, which might have somewhat different knowledge, attitude and behaviour in comparison with the general population. Thus, it is hardly possible to generalise to the general population of the study area. Hence, future community-based studies are recommended.

## Conclusion

Overall, alcohol consumption, tobacco smoking and unhealthy dietary practice were relatively high, whereas physical activity level was low among college students in Ethiopia. Additionally, knowledge of the behavioural risk factors of cancer is low. Therefore, awareness creation of a healthy lifestyle is important to reduce the risk of cancer. Moreover, a multi-sectoral collaboration of education, health, women’s affairs and sport sectors is needed for producing active and healthy students, and their combined actions could easily address the behavioural risk factors of cancer. Incorporation of healthy life styles and other cancer control measures in the curriculum of non-healthy students might help to reduce cancer burden. Furthermore, restricting unhealthy foods from family food dishes and college compounds could help to reduce the consumption of unhealthy foods and give value to healthy foods. Furthermore, increasing the production and access of healthy foods are important to maintain the year-round availability and regular consumption of healthy foods. In conclusion, policy formulation on cancer prevention and control could help to organise activities, strength multi-sectoral collaboration and encourage stakeholders to perform their duties. As mentioned above, a high magnitude of behavioural risk factors of cancer was observed, which emphasised the necessity of text message trials for the prevention of cancer in young and productive age groups.

## List of abbreviations

ED, Every day; FSW, Five to six times per week; IQR, Interquartile range; LMICs, Low- and middle-income countries; NC, Never consumed; NCD, Non-communicable disease; OW, Once per week; OPM, Once per month; TFW, Three to four times per week; TW, Two times per week; SD, Standard deviation; WHO, World Health Organization.

## Declarations

### Ethical consideration

The protocol of this study obtained ethical approval from the Institutional Review Board of Wollo University, College of Medicine and Health Sciences. Written consent was obtained from each participant after an explanation of the study purpose, description of possible risks and benefits. Honest explanation of the study purpose, description of the benefits and an offer to answer all inquiries made to the respondents. Additionally, affirmation was given that they are free to withdraw or discontinue participation without any form of prejudice. Privacy and confidentiality of collected information were ensured throughout the process; measures were taken to ensure respect, dignity and freedom of each individual participating in the study. The study was following the principles of the declaration of Helsinki.

### Consent for publication

Not applicable.

### Availability of data and materials

Available on request.

### Competing interests

The authors declare that they have no competing interests.

### Funding

Partial funding for data collection only was obtained from Wollo University.

### Author contributions

FMH designed the study, collected the data and wrote the original draft. HYH contributed to data analysis and interpretation, validation and write-up of the manuscript. Other authors contributed to data interpretation, resource acquisition, reviewing and editing. All authors read, critically revised and approved the final manuscript.

## Figures and Tables

**Table 1.** Sociodemographic characteristics of students in Dessie public colleges, 2021 (*N* = 200).

Variable	Frequency	Percent
**Sex**		
**Male**	162	81.0
**Female**	38	19.0
**Age group (years)**		
**18–24**	164	82.0
**25–30**	31	15.5
**31–35**	5	2.5
**Religion**		
**Muslim**	109	54.5
**Orthodox**	91	45.5
**Ethnicity**		
**Amhara**	378	89.4
**Tigre**	24	5.7
**Oromo**	19	4.5
**Others**	2	0.5
**Marital status**		
**Never married**	175	87.5
**Currently married**	15	7.5
**Divorced**	4	2.0
**Widowed**	3	1.5
**Separated**	3	1.5

**Table 2.** Cigarette smoking and alcohol consumption among students in Dessie public colleges, 2021 (*N* = 200).

Variable	Frequency	Percent
**Currently smoking**		
**Yes**	32	16.0
**No**	168	84.0
**Daily smoking**		
**Yes**	30	93.7
**No**	2	6.3
**Type of smoker**		
**Light**	22	68.8
**Medium**	8	25.0
**Heavy**	1	3.1
**Very heavy**	1	3.1
**Age onset of smoking**		
**Unknown**	15	46.9
**10–15**	5	15.6
**16–20**	12	37.5
**Ever smoker**		
**Yes**	14	7.0
**No**	182	91.0
**Smokeless tobacco**		
**Yes**	19	9.5
**No**	181	90.5
**Passive smoker**		
**Yes**	19	9.5
**No**	181	90.5
**Ever alcohol drinking**		
**Yes**	61	30.5
**No**	139	69.5
**Alcohol drinking within a month**		
**Yes**	55	90.2
**No**	6	9.7
**Drinking frequency/week**		
**Light (<2)**	13	23.6
**Moderate (2–4)**	19	34.6
**Heavy (5+)**	23	41.8
**Drinking with meals**		
**Usually**	11	20
**Sometimes**	9	16.4
**Rarely**	15	27.2
**Never**	20	36.4

**Table 3.** Physical activity of students in Dessie public colleges, 2021 (*N* = 200).

Variable	Frequency	Percent
**Vigorous physical activity**		
**Yes**	86	43.0
**No**	114	57.0
**Vigorous activity/week**		
**1–2 days**	28	32.6
**3–-4 days**	26	30.2
**5–7 days**	32	37.2
**Duration of vigorous activity/day**		
**1–2 hours**	67	77.9
**3–4 hours**	12	14.0
**5–7 hours**	7	8.1
**Moderate activity**		
**Yes**	81	40.5
**No**	119	59.5
**Moderate activity/week**		
**1–2 days**	27	33.3
**3–4 days**	37	45.7
**5–7 days**	17	21.0
**Duration of moderate activity/day**		
**1–3 hours**	69	85.2
**4–6 hours**	5	6.2
**7–10 hours**	7	8.6
**Walking/using bicycle**		
**Yes**	101	50.5
**No**	99	49.5
**Walking/using bicycle/week**		
**1–2 days**	30	29.7
**3–4 days**	26	25.7
**5–7 days**	45	44.6
**Duration walking/bicycle/day**		
**1–3 hours**	83	82.2
**4–6 hours**	4	4.0
**7–10 hours**	14	13.8
**Duration of sitting/day**		
**1–3 hours**	134	67.0
**4–6 hours**	34	17.0
**7–10 hours**	8	4.0
**>10 hours**	22	11.0
**Sleeping duration/day**		
**1–3 hours**	8	4.1
**4–6 hours**	48	24.5
**7–10 hours**	127	64.8
**>10 hours**	13	6.6

**Table 4.** Dietary habit of students in Dessie public colleges, 2021 (*N* = 200).

Variable	NC (%)	OPM (%)	TTM (%)	OW (%)	TW (%)	TFW (%)	FSW (%)	ED (%)
**Cereals**	-	-	-	-	14 (7.0)	12 (6.0)	9 (4.5)	165 (82.5)
**Legumes**	-	-	-	33 (16.5)	30 (15.0)	11 (5.5)	31 (15.5)	95 (47.5)
**Nuts and Vegetable oils**	29 (14.5)	16 (8.0)	29 (14.5)	22 (11.0)	13 (6.5)	25 (12.5)	21 (10.5)	45 (22.5)
**Fruits**	19 (9.5)	35 (17.5)	62 (31.0)	36 (18.0)	6 (3.0)	15 (7.5)	6 (3.0)	21 (10.5)
**Vegetables**	8 (4.0)	35 (17.5)	57 (28.5)	34 (17.0)	36 (18.0)	7 (3.5)	10 (5.0)	13 (6.5)
**Flesh and organ meat**	23 (11.5)	45 (22.5)	51 (25.5)	40 (20.0)	11 (5.5)	19 (9.5)	3 (1.5)	8 (4.0)
**Fish and sea foods**	118 (59.0)	55 (27.5)	27 (13.5)	-	-	-	-	-
**Eggs**	34 (17.0)	72 (36.0)	33 (16.5)	24 (12.0)	22 (11.0)	8 (4.0)	2 (1.0)	5 (2.5)
**Dairy products**	23 (11.5)	76 (38.0)	30 (15.0)	28 (14.0)	11 (5.5)	7 (3.5)	14 (7.0)	11 (5.5)
**Street foods**	38 (19.0)	29 (14.5)	11 (5.5)	40 (20.0)	25 (12.5)	16 (8.0)	5 (2.5)	36 (18.0)
**Sugars and sweets**	19 (9.5)	38 (19.0)	36 (18.0)	37 (18.5)	30 (15.0)	15 (7.5)	16 (8.0)	9 (4.5)
**Salt**	-	-	28 (14.0)	23 (11.5)	21 (10.5)	17 (8.5)	14 (7.0)	97 (48.5)
**Fried and pre-packed snacks**	9 (4.5)	14 (7.0)	39 (19.5)	35 (17.5)	20 (10.0)	19 (9.5)	24 (12.0)	40 (20.0)
**Butter, fat and palm oil**	26 (13.0)	43 (21.5)	29 (15.5)	33 (16.5)	27 (13.5)	14 (7.0)	14 (7.0)	14 (7.0)
**Coffee and tea**	6 (3.0)	30 (15.0)	31 (15.5)	17 (8.5)	36 (18.0)	32 (16.0)	15 (7.5)	33 (16.5)

**Table 5.** Cross tab results of behavioural factors with sex in public colleges, Ethiopia, 2021.

Variable	Male *N* (%)	Female *N* (%)	*p*-value
**Age group (years)**			
**18–24**	136 (84.0)	28 (73.7)	
**25–30**	26 (16.0)	5 (13.2)	
**31–35**	0	5 (13.2)	
**Knowledge**			
**Poor**	100 (61.7)	22 (57.9)	0.66
**Good**	62 (38.3)	16 (42.1)	
**Dietary habit**			
**Poor**	83 (51.2)	21 (55.3)	0.65
**Good**	79 (48.8)	17 (44.7)	
**Smoking**			
**Yes**	29 (17.9)	3 (7.9)	0.14
**No**	133 (82.1)	35 (92.1)	
Alcohol drinking			
**Yes**	50 (30.9)	5 (13.2)	0.02
**No**	112 (69.1)	33 (86.8)	
**Vigorous physical activity**			
**Yes**	79 (48.8)	7 (18.4)	0.001
**No**	83 (51.2)	31 (81.6)	
**Moderate physical activity**			
**Yes**	69 (42.6%)	12 (31.6%)	0.21
**No**	93 (57.4%)	26 (68.4%)	
**Sitting duration/day**			
**1–3 hours**	114 (71.2)	20 (52.6)	
**4–6 hours**	24 (15.0)	10 (26.3)	
**7–10 hours**	8 (5.0)	0	
**>10 hours**	14 (8.8)	8 (21.1)	
**Sleeping duration/day**			
**1–3 hours**	5 (3.2)	3 (7.9)	0.15
**4–6 hours**	36 (22.8)	12 (31.6)	
**7–10 hours**	108 (68.4)	19 (50.0)	
**>10 hours**	9 (5.7)	4 (10.5)	
